# Selections and Abstracts

**Published:** 1905-09

**Authors:** 


### SELECTIONS AND ABSTRACTS.

---

### An Abstract of the Presidential Address on The American Medical Association: Its Origin, Progress, and Purpose.

*Delivered at the Fifty-Sixth Annual session of the American Medical Association at Portland, Oregon, July 11-14, 1905.*

By Lewis S. McMurtry, A.M., M.D.,
Louisville, Ky.

[After referring to the purposes and advantages of such medical gatherings; a felicitous congratulation on the fact that "perfect harmony prevails;" "the differences which at times have divided us have all been satisfactorily adjusted," and a reference to the year's dead, with special mention of Dr. Nathan S. Davis and Dr. B. C. Pennington, the speaker rapidly traced the origin and evolution of the Association. In reference to the work of the sections he repeated as a result of his own observation and experience, the recommendation of his predecessor in the chair, that the secretary of each section should be elected for a term of years. "No national society," he said, "can maintain its efficiency which changes its secretary annually." He further recommended that the section officers should meet as soon as possible after the adjournment of the session, to formulate definite plans for the next year's scientific work. To such action last year was to be attributed the admirable scientific programme of the present meeting.

The President then eulogized the Association's *Journal*, and paid a well-merited compliment to Dr. Simmons: "By the untiring daily services of the present able editor, it has leaped into the very front rank of scientific publications, and in all that a great weekly medical journal should be it has no superior in the world."

Describing the *New Era*, the speaker continued]:

At the annual session of 1900 a committee on reorganization was appointed, and one year later, at St. Paul, the report of the committee was submitted and adopted. It included a new constitution, which altered the basis of apportionment for delegates, so as to reduce the delegate body to 150, and definitely established a close relationship between the national organization and the State, district and county societies. For the first time a practical scheme of complete organization of the medical profession of the United States was provided. This is the fourth annual session held under the new plan of organization.

Previous to the reorganization, the valuable scientific work of the sections constituted almost the sum total of the effective work accomplished by the Association. Matters appertaining to medical education, and to the welfare of the profession received no deliberate consideration and in consequence no decisive action was carried out. This condition existed for the reasons already mentioned. In a word, the very objects and aims for which the Association was organized were thwarted by the growth of the Association into an unwieldy delegate body. Under the reorganization, the House of Delegates, in which the membership of the State societies is proportionately represented, now gives deliberate consideration to those important matters, already indicated, which under the former organization were neglected.

The influence of the revised plan of organization was immediately apparent in the increased attendance at the annual sessions, and the stimulus felt in every purpose of the Association. And each year this influence has grown, until some idea can now be formed of the great possibilities to come from organization on a definite and practical plan. The good results to accrue to the profession as a whole, and to every member as an individual, are so positive that no subject can deserve more careful consideration by this body than that of medical organization. Indeed, it is the fundamental question before us, and on its decision depend the results of all our other efforts in all directions.

The necessity of reorganization was appreciated by the leading members of the Association long before it was accomplished. In 1888 Dr. N. S. Davis, as chairman of a committee appointed for the purpose, reported a scheme of reorganization very similar to that adopted in 1901 at St. Paul.

ORGANIZATION.

"The object of this association shall be to federate into one compact organization the medical profession of the United States, for the purpose of fostering the growth and diffusion of medical knowledge, of promoting friendly intercourse among American physicians, of safeguarding the material interests of the medical profession, of elevating the standard of medical education, of securing the enactment and enforcement of medical laws, of enlightening and directing public opinion in regard to the broad problems of State medicine, and of representing to the world the practical accomplishments of scientific medicine." (Article II, Constitution of the American Medical Association.)

If every physician worked alone, relied on his own unaided observation for his knowledge, never looking outside of his own scope of view, ignorance would prevail and there would be no progress in medical science. Medicine is not an exact science, and until perfected by extorting from Nature all her secrets, it must from the nature of things continue to be a progressive science. Never before in the history of medicine has such marked progress in all its departments been made as during the present age. Theories have been supplanted by facts; laboratory research and clinical investigation have taken the place of tradition and authoritative opinion. To rely on the accomplishments of the college period is to be left behind in hopeless incompetency. The advance of medical knowledge is to be observed first in our medical societies, and afterward in our scientific medical journals. In the medical societies innovations are subjected to criticism and discussion by those competent to judge the merits of scientific contributions. Moreover, there is a stimulus to study and investigation from association with workers in the same field; and one obtains a broader view of every subject so considered. The physician, more than any other professional man, is isolated by the conditions of his life, and to no profession is the educating influence of society work so essential and invaluable. This same condition of isolation is at the foundation, for the most part, of the jealousies and petty bickerings so prevalent in our profession. These troubles are, as a rule, the result of misunderstandings, and are both prevented and corrected by coming together in a society composed of physicians. The lonely worker in any calling is prone to become narrow, suspicious and morbid. Our medical societies are the great post-graduate schools of the profession, where knowledge is increased and individual character devleoped.

But the promotion of scientific investigation, and the diffusion of medical knowledge are not the only objects of organization. Our profession has a most essential and important duty in relation to the public health. Through no other agency can municipal, State and national health authorities formulate and secure recognition of laws to prevent and control disease. Another duty no less essential is so to regulate and control medical education that the ignorant and unworthy shall not be admitted to the privileges of the profession, thereby preserving the time-honored standard of professional honor and scientific capability. In this age of organization, without it the profession is powerless to secure the enactment of humane sanitary laws, by which alone the people can be protected from preventable diseases. The welfare of the profession, collectively and individually, can only be subserved by organization. Without such organization our profession, as a body politic, will be without unanimity of sentiment or action in relation to the important scientific, ethical, and social questions which confront us; and consequently without influence politically, socially or otherwise.

In the declaration of the constitution adopted at St. Paul, which I have just quoted, the American Medical Association has undertaken to place within reach of every reputable medical practitioner in the United States these incalculable advantages of medical organization.

The Committee on Reorganization appointed in 1900 has treated this subject in a masterly manner. After a careful study of the condition of the profession throughout the States with relation to organization, reports have been made to the Association showing the needs of the profession and the extent of the work required. A uniform plan of organization for State and county societies, making the county society the unit of organization, and federating all State societies in the National Association in harmonious cooperation, has been prepared by the committee. The able chairman of this committee has with remarkable tact, patience, and good judgment given his personal supervision to this great work in almost every State and Territory. In his latest report it is announced the States and Territories, except three, and including Hawaii and Porto Rico, are now organized on a practically uniform plan, with universal local societies, and coincident membership in them and the State associations as the cardinal feature. To illustrate the magnitude of this work, I mention that, under the stimulus of reorganization after the plan of the committee, the Michigan State Medical Association increased in membership in one year from 452 to about 2,100. Texas increased the membership in the same time from 382 to 2,510, while several States quadrupled their membership. While these results are phenomenal and elicit our admiration, it will be realized how much remains to be done when it is considered that few of the States have over 50 per cent. of the eligible members of the profession enrolled as members of the society. Indeed, the work is yet almost in its infancy. What has been accomplished is an assurance for the future and, considering the brief time since reorganization began, is a high tribute to the enterprise of the National Association and the work of its able and efficient committee. The possibilities of this work are stupendous, and as it proceeds the Association can confidently undertake the great reforms of such incalculable moment to the profession and the public. In his official report to the House of Delegates one year ago, the chairman of the Committee on Organization, after reporting the splendid results of his labors during the year, said:

> The real test of our organization will come in each State when the first outburst of enthusiasm has passed, and county societies, the foundation for everything, are likely to disappear as rapidly as they have been formed, unless their usefulness to the rank and file of the profession can be demonstrated in a very broad way.

In recognition of this fact, it is of the utmost importance that the personal attention so effectively given to this work throughout the States by the chairman of the committee be continued.

MEDICAL EDUCATION.

When the American Medical Association was founded in May, 1846, the delegates assembled in response to the following preamble and resolutions of the Medical Society of the State of New York :

WHEREAS, It is believed that a national convention would be conducive to the elevation of the standard of medical education in the United States, and

WHEREAS, There is no mode of accomplishing so desirable an object without concert of action on the part of the medical societies, colleges and institutions of all the States; therefore, be it

*Resolved*, That the Medical Society of the State of New York earnestly recommends a national convention of delegates from medical societies and colleges of the whole Union, to convene in the City of New York, on the first Tuesday in May, in the year 1846, for the purpose of adopting some concerted action on the subject set forth in the foregoing preamble.

From this it is apparent that this association was founded with the avowed and specific purpose of elevating the standard of medical education in this country. This subject has constantly received consideration from the association throughout the years of its existence; and while no distinct and positive reform marks its action, the perpetual demand for improvement undoubtedly moulded the public sentiment of the profession which ultimately stimulated action on the part of the medical colleges. Since the addresses of my two distinguished predecessors, who immediately preceded me in this chair, were devoted to the consideration of medical education, I shall only make brief allusion to this subject. The several national organizations and the many State boards which are now dealing with this important professional problem invest the changing conditions with peculiar interest. The present is a transition period in the advancement and reform of medical education in America.

The consideration of this subject in the House of Delegates culminated in the establishment last year of the Council on Medical Education of the American Medical Association. During the past year the council has taken up its work with exceptional energy and good judgment, and is directing its efforts along most practical lines. The first annual conference was held in Chicago April 20th last. Representatives of many State examining and licensing boards of the American and Southern Medical College Associations, and of the government medical services, attended and participated in the discussions of the papers presented. The plan and scope of the work of the council can be best appreciated by a consideration of this extract from the address of the distinguished chairman of the council delivered to the conference:

"What we need is co-operation, especially the co-operation between the medical profession, represented by the American Medical Association, and the State and county medical societies, and the State authorities, represented by the State licensing and examining boards. The most important question, therefore, before this conference is, How can the American medical profession and the State licensing bodies co-operate to elevate and control medical education? It is believed that such co-operation is possible. In such co-operation it will be the function of the American Medical Association to represent and possibly mold the opinion of the medical profession and employ its influence and the influence of the county and State medical societies in obtaining proper medical legislation. In such co-operation it will be the function of the State licensing bodies to protect the interests of the public and the profession by seeing that the medical laws are properly interpreted and enforced, and from their intimate knowledge with the medical acts they can often be of service in securing and modifying medical legislation.

"It is not the purpose of the Council on Medical Education of the American Medical Association to attempt to arrogate to itself any special powers, nor does it desire to criticise or interfere in any way with any of the agencies which are already in the field. If its creation is to result in good, it must be the means of obtaining co-operation between the medical profession, and the medical schools, the colleges of arts, the State examining boards, the government services, and all the factors which are interested in elevating and controlling medical education."

By securing co-operation of the various State and national bodies engaged in this important work, recognizing the varied interests involved in the numerous States of the Union, and fostering an intelligent public sentiment, so essential for every great reform, the influence of the Association will be of the most effective character.

### MEDICAL LEGISLATION.

Chapter VII, section 3, of the by-laws provides a permanent Committee on Medical Legislation "to represent before Congress and elsewhere the wishes of this Association regarding any proposed legislation that in any respect bears on the promotion and preservation of the public health, or on the material or moral welfare of the medical profession." This committee was appointed in 1903, and under the leadership of its accomplished and energetic chairman proceeded at once to organize the auxiliary committee provided in the by-laws. This being a permanent committee, its work will be continued from year to year. With the auxiliary committee, representing every State in the Union, and the several medical departments of the government service, every representative of the people in Congress can be reached directly. The importance of this work is so apparent that I need only call attention to it at this time and urge on all members of the Association the necessity of aiding and co-operating with the committee at all times. The disregard of the good work of the medical profession in the public service (to say nothing of the indignities offered) on the part of Congress is notorious; and it is only by such concentrated power of the profession, intelligently directed, that such injustice may be overcome. I commend to your consideration the report of this committee in order that you may appreciate the important work already projected.

COUNCIL ON PHARMACY AND CHEMISTRY.

The use of proprietary medicines in the treatment of diseases has become one of the most confusing and demoralizing questions of the day. All proprietary medicines must not be classed as secret nostrums, for there are many honestly made and ethically advertised proprietary preparations that have therapeutic value and that deserve the approval of the medical profession. But there are many preparations offered the profession, which are protected by copyright or trade-mark, with formulae more or less fictitious, and for which are made extravagant claims, which are in fact secret remedies. These preparations are so exploited by the manufacturer that the physician is persuaded to use them instead of writing a prescription; and since they usually bear popular names and plausible therapeutic claims, they appeal to the fancy of the laity. The field is an enticing one for commercial enterprise since these preparations in many instances are simple mixtures and contain the most inexpensive drugs. The use of such remedies is both unscientific and unjust, alike to physician and to patient.

The separation of the legitimate pharmaceutical preparations from the class of fraudulent nostrums described is a most difficult undertaking. This perplexing problem forced itself from year to year on the attention of the board of trustees in the effort of the board to keep the advertising pages of the *Journal* free from unethical advertisements. In order to have thorough protection and to make no unjust discrimination, the board has established the Council on Pharmacy and Chemistry to make the necessary investigations. The council is composed of pharmacists and chemists of national and international reputation. It will be the aim of the council to publish in book form a list of the preparations which are not official, yet which conform to the proper ethical standard. The work of the council will be similar to the Committee on Revision of the United States Pharmacopoea.

The magnitude and importance of this work is such that I desire to direct attention to it here, and to commend it to the members of the Association as deserving every possible aid and advancement. It is the only practical way to deliver the profession from one of the greatest curses that ever came to it.

It has been my endeavor on this occasion to outline the plans of the founders of the association, briefly to trace the evolution of those plans throughout half a century of progress, and to recount some of the purposes that invite our active exertions at the present time. From a small body of delegates our association has increased until it is now the largest medical organization in the world. It owns valuable property, has accumulated a considerable fund, and has a large annual income, all of which belongs to, and is subject to the control of, the members who through their delegates select the Board of Trustees to manage their funds.

The possibilities of the work before us are almost beyond calculation. In acting as the representative and agent of the 120,000 physicians of the United States, the association is assuming great responsibilities, which will increase from year to year. It will require administrative and executive ability of the highest order to meet these demands, but there is both prophecy and proof in the work already accomplished that men will appear as needed to discharge the supreme duties of a great profession in behalf of science and humanity.—*St. Louis Medical Review.*

### THE DECISION TO OPERATE.*

BY A. M. CARTLEDGE, M.D., LOUISVILLE, KY.

When your worthy president did me the honor to invite me to deliver an address upon surgery, I felt honored indeed, and promptly accepted; but when the time arrived to furnish the title of my discourse, I was sorely puzzled. Years ago it was customary in such addresses to review the most important contributions on the subject which the previous year had brought forth. Later, appendicitis, or some equally burning question in intra-abdominal pathology, formed the text. Although the usual subjects have been worked overtime, it is a great relief to know that whatever one says is non-debatable—that is, where the author can hear it. This last is one of its most attractive features.

The broad title, "The Decision to Operate," allows one the privilege, not only of reviewing current surgical events, but to indulge in a heart-to-heart talk between surgeon and physician, and surgeon and surgeon. We all have our fads and our convictions; some of these are the result of reading, some of teaching, and some of experience. While I have little new to offer you, I would beg to say that the deductions set forth are the result of experience, and if some of them impress you as being fads I hope you will attribute it to this fact.

Above all things, I would impress upon both physician and surgeon the eternal fitness of time and opportunity as they relate to surgical operations. This is the day of operations; the laity is keenly alive to it; it is interesting the press, the pulpit, and the people more than we appreciate. Surgical operations, or "the knife," as the youthful journalist is so fond of styling it, is, next to the getting of riches, uppermost in the human mind at the present time. Politics, international wars, the doings of kings, the achievements of science, especially in practical electricity, all claim more space in the daily press, but are less talked of than surgical operations in the private circle of social life. There is still left a remnant of the modesty which once caused people to refrain from

*Address in Surgery before the Mississippi Valley Medical Association, Memphis, Tenn., October 3, 1903.

discussing their physical afflictions, otherwise the doings of our profession would be offensively notorious in the daily press. There is scarcely a day that the busy doctor does not hear such remarks as these: "The operation was done too late," "The doctor should not have operated at all," "The operation caused the patient's death," etc.

Such remarks lead us to ask, "Is the laity ever right as to a surgical or medical proposition, or any other proposition of a technical kind requiring for its elucidation special knowledge?" I answer unhesitatingly, "Yes." It is the exaction born of a desire for the betterment of their physical well-being, that has made our work so glorious to-day. That we are criticised none can deny; that such criticism is often most harsh and unjust none can dispute, but is a necessity and sometimes it is just, and furnishes the real stimulus to progress.

The family physician, when criticism is rampant, usually comes in for the largest share. At first thought this touches our deepest vein of pity, for is it not he who bears the brunt of the battle from the mother's womb to the grave? Sunshine and rain, comfort and cold are alike to him, so great is his affection for the man, woman or child he has watched through all the years of their existence. How often he has been the comforting angel; how often brought relief from pain and suffering, but alas! he did not recognize the last case soon enough; the surgeon was called in too late; it is too late, the poor old doctor is undone; the beautiful confidence once imposed in him, and which was the light of his soul, the mainstay which made his great burden seem light, has gone; past benefactions are forgotten. In the bitterness of his sorrow he says: "What I thought was a warm and beauteous world is a cold, ungrateful habitation!" But this is evolution, and lucky, indeed, is the man who can so plan and meet the requirements of his environment as to escape criticism.

Let us take a general inventory of the methods of our work and see if we do not deserve some of the criticism heaped upon us. If so, how can we avoid, at least in part, the unpleasant comments upon our professional work?

Undoubtedly the first great responsibility in deciding that an operation should be performed rests with the physician, usually with the family physician, who is keenly alive to alarm and perturbation his announcement will cause, to both patient and friends. Therefore, he should be prepared to make such announcement with the greatest clearness and candor, mingled with as much encourment and hope as the condition will justify.

Permit me right here to sound a note of warning against the too common practice of treating surgical operations as trivial affairs, free from danger and pain. While the results of modern surgery have so far improved as to make the danger almost nothing compared to that of a few years ago, still there is an unknown quantity in every surgical operation, and the case in point may be the very one in which it will assert itself in a most disastrous way. In deciding upon an operation and recommending the same to the patient, we can not do better than to state the mortality of such operations when performed by skillful surgeons. It is an excellent practice to say to the patient, or those entrusted with his welfare: "If I should operate upon a hundred people afflicted as you are, eighty-six (or ninety-eight as the case might be) would recover, and fourteen (or two) would die." They fully understand such a statement, and, while it may sound a little cold and unfeeling, it will usually give more comfort and reassurance in the end than the unfortunate and usual plan of saying: "Oh, there is no danger whatever!" The most ignorant people know better; they have had friends and perhaps relatives who died following surgical operations. Physicians and surgeons do not talk this way to deceive the patient, but from a mistaken idea that it gives encouragement and is a kindness. Remember, it is not our function to persuade people to be operated upon, but rather to honestly diagnose their ailments and give an honest opinion and advice as the result of our examination.

Never assume the role of begging people to be operated upon. This practice leads to a great deal of trouble if persisted in. I have known of more than one instance where the family physician assured the patient there was no danger in the operation; the patient accepted his statement and the result was death. Now, the people do not view such a case from our standpoint, and the doctor was acting from a mistaken idea of what was necessary in order to get the patient to do what we will grant was the best for him to do. The verdict of the friends is short, and, taking the doctor's ante-mortem statement literally, highly logical: "The doctor was either a liar or a fool" (and there is little choice between the two).

I am convinced that some of the harshest and most uncompromising criticism by the laity comes from this practice. Tell the truth; tell it kindly; tell it encouragingly, but remember, no one has a better right to know the dangers of a surgical operation than the unfortunate victim about to undergo one. Instead of deterring people from having operations performed, such a course gives them confidence, for they feel they know all—nothing is kept from them.

In this day, when Dr. A. reports his hundred operations for this, and Dr. B. his thousand operations for another trouble, the busy physician, as he scans his weekly or monthly medical journal, concludes that nearly everything is amenable to speedy aud certain cure by surgical intervention if he can only get his patient's consent. We must reckon on the patient's consent as a part of the decision to operate. Time and opportunity affect as much the outcome of surgical operations as methods, and have vastly more to do with the degree of criticism we are likely to be subjected to.

I wish to call especial attention to the decision to operate in affections confronting us almost daily, and the significance of time and opportunity. What I say is suggested chiefly by mistakes of others as well as some of my own. We are appalled at what seems the great increase of malignant disease, and operations for carcinoma are of almost daily occurrence with some of us.

As a broad proposition, modern surgery advocates the early removal by the knife, as giving the best results in the treatment of this dread disease. As a broad counter-proposition, the laity is convinced that the knife will not cure cancer. In answer to this wide difference of opinion we are accustomed to say that the laity only hears of the ones who die and not of the ones who recover. At any rate, the practical result is that the larger part of patients suffering from cancer do not try our remedy until everything else has failed and the disease is too far advanced to be benefited by surgical interference.

Are we doing what we should to better this deplorable state of affairs? Are we helping to bring the laity to view the question rightly? Have they not cause for refusing to believe our statements in regard to the treatment of cancer? Yes, or they will not do so. We deserve much criticism from this source.

To remedy matters it is not only necessary to urge early operation in malignant disease, but, what is equally important, learn to desist from operation in hopeless cancer cases. Such a course will soon make our position intelligible to the laity.

It is as much the province of science to know its limitations as as it is to know its capabilities. It is true ours is not an exact science, but it is rapidly approaching it, and these seemingly ignorant demands of the laity are of the greatest stimulus to that end. As a rule, we promise too much in even so-called favorable cases of malignant disease; and do entirely too many operations for utterly hopeless cases of carcinoma.

The knowledge and position of modern surgery can not afford to be longer disgraced by such lame and ignorant statements as, "Well, the patient is going to die, anyway, and it might cure him," made to the victim's relatives and friends. If the case is such a doubtful one it is better to state to the patient, or his relatives, that the prospects for permanent cure are most unpromising, but, after due deliberation, we believe we would advise operation as a possible relief, always remembering, if your diagnosis be correct and the disease has gotten to doubtful limitation, that the chances are ninety to ten that speedy recurrence will take place, and shape your statement in the decision to operate accordingly.

If for any reason, such as in cancer of the uterus, you should deem an operation advisable as a palliative procedure, make such an action clear to the family and friends. Protect yourself and your profession, as such a course will ultimately do the greatest good by preventing future sufferers from permitting themselves to go unrelieved until such a late and hopeless condition has been reached. The horror of pain, the fear of an anesthetic, and the dread of instruments, make a surgical operation to many people an ordeal little short of death. To such it is cruelty to advise operation in utterly hopeless cases of malignant disease, even for the sake of palliation. People view the knife as radical, curative, though fearful, and do not take much stock in it as a palliative measure. While for a time they may agree with you that the poor victim would have died anyway, later, as they sorrow for the departed loved one, they regret having consented to the operation, and wish, as they express it, they had let the unfortunate one "die in peace."

In cancer of the uterus, if seen very early, we should advise complete removal of the organ at once, yet promose very little as to return, for there is no chapter in the history of malignant disease as gloomy as this. The permanent cures are so few, even when the operation is performed in the first stages of the disease, that they leave little for encouragement.

Cancer of the breast is more hopeful, but hopeless enough. Physicians are not sufficiently impressed with the value of time in these cases. We find them frequently watching a breast tumor for a year before deciding upon consultation, or advising operation. Just remember that 85 per cent. of such tumors are malignant, and that three months may permit of hopeless extension beyond operative benefit, and you will be impressed with your grave responsibility. Every case of breast tumor should be explored at once, except those where the disease is so extensive as to be obviously inoperable. My experience justifies me in saying that if we avoid operation upon what every experienced surgeon knows to be hopeless cases of cancer of the breast, and to immediate and widely searching operations upon the more favorable ones, the people will soon be convinced of the lasting value of early operation for this trouble.

In cancer of the maxillary bones I believe it is sound practice to advise operation in almost every case affecting the lower jaw, and to refuse operation in almost every case involving the superior maxilla. Next to the uterus I do not know of a situation in the body where malignant disease is surer to return after operation than the superior maxilla. I am convinced that cancer of the alimentary canal is slower of growth and extension than is generally supposed, and that early operations promise much more than is usually accredited to them. This applies especially to the rectum and anus, wide excision by way of resection giving the happiest relief from an otherwise wretched death.

Be slow to decide upon operation in chronic brain troubles, such as suspected tumor. Exploring the brain is always serious; in fact, it is a most serious thing to take a little round piece of bone from the cranium, for it leaves a lasting hole in a very undesirable place. Brain surgery is most brilliant when it achieves its aim, which is rare, and most disappointing when it does not. Decide quickly upon operation in compressions and infections within the cranial cavity.

I know of no serious consequences that should cause you to advise immediate operation in serous and purulent accumulations of the pleura. However, it is unwise to wait until the fluid is pointing between the ribs, for it is almost impossible for the surgeon called in to escape the conviction that the family physician had overlooked the real condition, and is somewhat short in his physical diagnosis. If you really do discover fluid in the pleura do not spend weeks of time in endeavoring to cause its absorption, for, other than the small effusion in connection with acute surface pneumonia, these collections of fluid do not absorb.

Of all the diseases of the human body those occurring in the peritoneal cavity are the most difficult to diagnose, and most uncertain and irregular in the course they may pursue. It is little wonder that the busy general practitioner, with all the body's ills to think of, read about and look after, should encounter so much difficulty in deciding what to advise and when to advise in an acute disease involving the peritoneum. It is to him I would address this part of my discourse. Truly, his position in this day of surgical operations for acute abdominal disease is a most trying one. If he gives the wrong advice he is held up between the people on the one side and the surgeon who happens to be called later on the other. His position is perilous indeed.

In old times it was allowable for people to die of cramp colic, congestion of the bowels, inflammation of the bowels, and later of peritonitis. It is not so now, for this troublesome element we call the laity has been taught that the first two, namely, cramp colic and congestion, are almost myths, and that the last, peritonitis, when given as the cause of death, is the result of some trouble the doctor did not recognize.

Now, with probably thirty causes of intestinal obstruction and a half-dozen of acute peritonitis, the plot thickens, and truly this man with so much to do has his trials; yet, sad to relate, in many cases the tale is quickly told. If the busy doctor, especially in the country, will divest his mind of fine diagnostic points in acute abdominal troubles and settle down to a proper appreciation of two or three important symptoms, he will be better able to act promptly and wisely. Remember that the people are so far educated in most places as to know that accurate diagnoses of all acute abdominal diseases are impossible. But they have a right and do expect of us the ability to detect alarming symptoms and institute active measures, usually operative, to avert a fatal result. They do not demand to know that a rapidly extending peritonitis is the result of a perforating duodenal ulcer, of a perforated appendix, or a leaking pyosalpinx. What they are interested in is, can it be arrested?

Time is of more importance in acute peritoneal infection than in lesions elsewhere in the body, and truly, hours are golden here and the fate of many a life is decided during the peaceful sleep that ensues from a quieting hypodermatic injection of morphine. The great danger to life from peritoneal infection is right at the onset, during the first thirty hours. Usually the amount of damage done in this time will be enough to determine the outcome of the case If the patient survives forty-eight hours and does not show evidence of a fatally spreading infection, the disease will most likely result in recovery by subsidence or limitation to a local process. The wisest physician has no means of knowing, or even forming an opinion, as to the probable course and termination of an acute peritoneal infection; for, while we may by diagnostic skill locate and recognize the causative lesion, no man can estimate the degree of violence of the infectious material, or that other unknown quantity, the patient's resistance to infectious organisms. Then by acting promptly we avoid uncontrollable complications.

Very early operations in acute abdominal lesions of a suspected infectious nature are often truly preventive of a rapidly fatal issue.

Not to act is to be like a man who would not quench the beginning fire in his house, but rather wait to see if it would destroy the same. Nature makes the most alarming outcry as a signal of the fearful happenings likely to take place, as witness the agonizing pain of acute intestinal obstruction, or an acute distended and infected appendix. Or, again, the alarming shock of ruptured tubal pregnancy, or a perforating ulcer of the stomach. It usually requires no diagnostic skill to know these are not the manifestations of simple things. Stop but an hour and observe these warnings without masking them by overwhelming drugs which but deceive you, and lull the victim to a hopeless fate.

It is in such cases that the physician should exercise the boldest and best knowledge in the decision to operate. When in doubt as to the nature of an acute abdominal condition characterized by sudden pain or shock, decide upon operation and you will have few, if any, reasons to regret such action, but you will have many if you neglect to do so hoping to be able to make an accurate diagnosis. The patient and his friends would rather he would get well without a diagnosis than to die with one.

The happiest time to operate upon intestinal obstruction is when you first suspect the patient has one of the organic forms of this condition. I have had enough regrets in my early experience for not deciding upon immediate operation in intestinal obstructions to form an entire chapter of misery in the record of my professional work. It is so easy when boldly decided upon, so fatal when delayed. If, from circumstances over which we have no control, acute infections are not operated upon and nature seems to be successful in combating the process—that is, if after three, four or five days the pulse is holding its own and the inflammation gives evidence of becoming circumscribed—we should consider well the advisability of interference in attempts to better her work. Remember, the damage in most cases of infection of the peritoneum is done in the first fifty hours, and the fate of the patient is usually determined during that time.

There are two very sad thoughts in connection with the work in such cases: one is to think we have failed to act early and thus have prevented a fatal issue; the other is to think that later, by interrupting nature, we had a fatal issue, where, if she had been left undisturbed, her efforts might have been equal to tiding the patient over to a safely operative state.

And now, gentlemen, while I have told you little that you were not already familiar with, I feel that if I have succeeded in impressing you with the importance of time and opportunity as they relate to the decision to operate, these general remarks will not have been in vain.

---

### TREATMENT OF TYPHOID FEVER.*

BY GEORGE F. JEWETT, M.D., BRITTON, S. DAKOTA.

As it is the object of the physician to restore the previous physical condition, so it should be his aim to know and combat those conditions which cause or intensify the phenomena that go to make up the clinical history of disease. Until recent years the treatment of typhoid fever consisted solely of attacks upon its phenomena, after their development; now we seek also to prevent or limit their development. If the older plan of treatment were not still in vogue, there would be no excuse for this paper; that it is so is amply attested by current medical literature and mortality rates.

Though we now know the bacillary cause of this disease, we are yet unable to eradicate it. Though we know that the bacterial poisons are responsible for most if not all of the manifestations, we are yet to produce the serum or remedy that will counteract the effect of these poisons.

The object of this paper is to bring to the attention of this Society a plan of treatment that seems to me most worthy of adoption, pending the development of an efficient antitoxin; or, better still, the discovery of the agent that will destroy the bacillus within the host. This plan of treatment consists of antithermic, antiseptic and eliminative measures.

Since the advocacy of water in continued fevers by James Cur-

*Presented to the South Dakota Medical Society during its annual session at Deadwood, July 5–6, 1905.

rie, this agent has been applied externally by various methods for the reduction of temperature. Hot, warm, tepid, cold, iced water have all had their advocates. Having learned from my preceptor's practice that the sponge bath accomplished all the good, and was followed by none of the bad results of other external hydrotherapeutic measures, I have used no other. The choice of warm, tepid or cool water should be left to the patient. One portion of surface after another should be sponged while exposed to the air until it no longer feels hot to the attendant. The trunk will require more than the extremities. The head and neck may be kept constantly wet and evaporation accelerated by fanning if necessary.

There is one use of cold water not referred to in any of the standard works in my possession, that will reduce temperature and ameliorate the symptoms attending high temperature more rapidly than any other, and at the same time with less annoyance than any of those uses capable of producing prompt results. I refer to cold water enemata. The first mention of this procedure, as far as I can ascertain, was by Dr. Buchman, in the *Medical Record*, Sept. 28, 1889. I had then been using it for six years. He thinks that from one to three quarts of cold water can be easily and safely passed into the colon, which will rapidly lower a high temperature. He also claims for it the relief of tympanitic distention and the prevention of putrefactive changes in the bowel contents. As an example of what can be accomplished by this means, I will relate one of my first experiences with it in the fall of 1883:

I found the patient had been left in charge of his blind sister since morning, and it was now 3 p. m. Patient unconscious, muttering, picking at bed clothes, temperature 105°, could not be aroused. I immediately gave a cold water enema. Before I had finished, he opened his eyes, looked at me and said, "Good morning," from which time he was able to carry on conversation intelligently, though his replies were at first somewhat tardy. Before leaving him, I took the temperature again and found it 103°, two degrees less than before the injection.

The water should be allowed to enter the rectnm very slowly from a fountain syringe in order to favor its retention, but it fortunately turns out that the greater the urgency of the conditions demanding its use the less is the necessity for precaution to insure its retention. As much should be used as the patient will tolerate, and this amount will increase with the patient's experience. The injection may be repeated after three hours, if necessary, but it is not often necessary so soon. It has been found that, when needed twice a day, from 12 to 2 and from 6 to 8 p. m. give better results. Those must be exceptional eases in which the use of cold water in this manner will not curtail the external applications for heat reduction 50 to 100 per cent.

Heat reduction by this means is not to be accounted for by the heat capacity alone ol the water introduced. The water thus used becomes loaded with toxins as well; and by whatever avenue it may escape from the patient must escape also its load of toxins—thus influencing the temperature indirectly by removing a cause. If a bowel movement results, then is temperature still reduced by removal of causes in the shape of bacilli and fetid fecal matter. Besides, the presence of cold water in the lower bowel has the further effect ot lessening the congestion of the mucous membrane of the whole canal, thus favorably affecting the specific lesions.

As temperature is only a phenomenon of the disease, though so prominent as to furnish that part of its name that has not been supplanted, so the reduction of temperature is only a symptom treatment. So long as we can not eliminate the ultimate cause, may we not do better than *reduce* temperature? Can we not limit the production of fever by lessening its proximate causes? If we could sweep the bacteria from the alimentary canal or limit their multiplication we could suppress or limit their poisonous products—the chief if not sole cause of fever as well as the other phenomena of the disease. This can be done, I believe, by catharsis. Not only this, but by the use of hydragogue cathartics the hyperemia may be diminished and all the later lesions rendered of lesser gravity. This measure was one of those advocated by a Civil War surgeon in 1863, or thereabouts. His results were so favorable, covering a period of twenty-five years, that I was led to adopt his routine. He used the compound colcynth powders as a cathartic and spirit of nitrous ether as a diuretic or diaphoretic—in the latter dissolving quinine for its tonic effect. For several years I followed his routine with none but the happiest results. At the same time plenty of water was given, not only because it was necessary to the success of the remedies, but for its antipyretic effect, for which latter purpose it was used also externally and by rectum as required.

In my fruitless search for his article I came across the treatment of Dr. George M. Ramsay, surgeon Ninety-Fifth, New York, in the Medical and Surgical History of the War of the Rebellion, Part Third, Medical Volume, page 550. The medication was so similar, and the results so surprising that I quote his words: "To allay the fever give one grain of quinine in half a drachm of sweet spirits of nitre three or four times in twenty four hours. To restore the secretions and excretions use the following pill, one or more, or less than one daily, so as to obtain one movement of the bowels every twenty-four hours: A half grain each of iodide of mercury, ipecac and extract of hyoscyamus, and one grain each of camphor and compound extract of colocynth, with syrup as an excipient. The fever will abate, the tongue clean off and the appetite return within forty-eight hours after this treatment has been commenced."

"I adopted this treatment at Belle Plain, Va., in 1862-3, but before I had become fully satisfied of its potency I permitted a patient to sink into the typhoid condition; pulse 110, tongue dry as a chip, much swollen, black-brown in the center, concave on its dorsum and curled up at its edges. I gave him a dose of the solution of quinine in nitrous spirit and repeated it in fifteen minutes. In ten minutes more I gave him a third dose, and in five minutes after this last dose the tongue had become moist and rounded. Then I ordered tea and toast, of which he ate sparingly. Under the continued use of quinine and nitre drops three or four times daily, and the pill as described, this patient steadily improved and was returned to duty in ten days.

"Several cases of typhoid fever were treated in shelter-tents at Sharpesburg, Md., during very inclement, rainy and cold weather. Under the treatment as specified the cases terminated favorably in ten days.

"Again, in the winter of 1863, a most aggravated case was treated in the regimental hospital. The command had marched to Raccoon Ford, ten or twelve miles distant, and returned to its old camp the next day, where I found that this fever case had been without shelter from rain and cold for twenty-four hours, the quartermaster having taken down and carried away the hospital tent. As a result the patient had become much worse; he muttered and was incoherent; pulse 100, and weak. It was feared that he was beyond recovery; but, under the treatment described, his tongue became moist and clean in forty-eight hours, and convalescence progressed rapidly. After twenty years of civil practice I continue to place implicit reliance on this method of treatment."

I am aware that these statements will appear extravagant to many, but, with my own experience with the routine above referred to, so nearly identical, I do not believe he has overdrawn his account. Patients very soon began to complain of the quinine-niter solution, and this complaint was an infallible indication of an all-round improvement.

The fear of aggravating the local lesions and causing hemorrhage by a rational administration of cathartics in typhoid fever certainly has found no foundation in experience. Some recognized leaders in the profession say they never give cathartics and advise against their use. It seems to me that the advice of one who never did is not worth much. Graham, in Sajous' *Cyclopedia of Practical Medicine*, says, from personal observation: "That, in a number of cases, the temperature falls to normal or nearly so within twenty-four hours after free purgation is induced; second, that cases at the end of the second or in the third week, when the patients are in a low toxemic condition—as shown by the dry cracked tongue, sordes, low, muttering delirium, and meteorism—have experienced decided and favorable change within twenty-four hours after free purgation. The writer is quite aware that such practice may possibly tend to the onset of hemorrhage, but he considers the dangers from the latter to be less than from the poisoned condition of the system."

He says that he is aware that such practice *may possibly* tend to the onset of hemorrhage. That such is not the case is proven by the extreme rarity and mildness of hemorrhage in cases to whom cathartics are given as compared with cases not so treated. I have not only continued the treatment in the event of the slight hemorrhages that have occurred under it, but have, in consultation, advised its adoption in cases with considerable hemorrhage, and have never had occasion to regret doing so. Whatever danger may be impending will certainly be diminished by depletion of the wall and lumen of the gut, and the muscular activity involved is just as rationally desired in case of hemorrhage as in other organs where such action is invoked for its arrest.

When salol came into use as an intestinal antiseptic I added it to my routine, but could not see that any better results were obtained. I found that tablets and capsules of salol passed without apparent loss, and this led me to believe the crystals did the same. This was due, no doubt, to the cathartics.

When the Woodbridge remedies were put upon the market I dropped other cathartics as well as my quinine-niter solution, and began the use of tablet No. 2, which I have used ir every case I have had since. These are given every hour at first. As soon as they may be given less frequently and still keep the bowels active, I begin the use of guiacol carbonate alone or with turpentine, eucalyptus and thymol.

I have never omitted the rectal injections of cold water except in those cases seen early enough to be aborted by purgative and antiseptics.

As to diet, it has been my rule to allow what was desired by the patient. If asked to advise, I suggested well-cooked gruels, eggs rare or thoroughly done, milk with a cereal to prevent large curds from forming, dried beef to be chewed until only a white string is left, and that spit out, and last, but best of all, buttermilk. Only once did I have occasion to regret the laxity of my rule when three patients in the same house had an abrupt rise of temperature after partaking of fish. Once I hesitated at the patient's request, when she would have kraut or nothing. The patient was my landlady, and tired me with her thrice daily importunities until I told her to eat it, but that she must assume the consequences. She did eat it, and progressed finely.

Treatment of typhoid fever along these lines has been so gratifying that nothing remains to be desired but some agent of which one many administer one dose, and, presto change, the patient is well. When patients are placed under the treatment outlined during the first week, the typhoid state and complications become an unknown quantity and the duration is shortened by half if not actually aborted in a few days. At any stage of the disease the patient may be put upon this treatment with immediate advantage, except in the solitary event that makes the case one for the surgeon or undertaker.

---

### INFLUENZA: ITS SEQUELÆ AND THERAPEUTICS.

BY C. B. McANALLY, MADISON, N. C.

It might not be out of place in the beginning of this paper to give a brief definition and history of this disease. Under a variety of names, Russian fever, epidemic catarrh, lagrippe, and influenza, the disease has been well known for more than two centuries. An infectious disease with a continued form of fever, accompanied by a great prostration of all the vital powers, with an almost universal tendency to inflammatory conditions of one or both of the respiratory tract and alimentary canal. During the past seventy-five years we have had four epidemics in this country. The first in 1830, and the last reaching North Carolina in 1891. After each epidemic, for a period of two to five years, we have had it in almost an epidemic form each year, gradually becoming less frequent and less severe. It is one of the most contagious diseases known and is transmitted directly from person to person, spreading over whole nations and even continents within a few weeks. The bacillus, or causative factor of the disease, was isolated by Pfeiffer—and contrary to the usually accepted theory, is found in more than five per cent. of all acute tonsillar and laryngeal affections, thus accounting for the isolated cases we so often see each year, far removed from the epidemics themselves. One very high authority has recently declared that it is highly improbable that we ever have it or see it in an endemic form. Yet I am sure many of the gentlemen before me to-day share my experience, in that they see a few cases each year. The importance of knowing what Auerbach has established in regard to the influenza bacilli being found endemically in such a large per cent. of acute throat troubles, can not be overestimated, inasmuch as it renders absolutely certain the diagnosis we so often make in these isolated cases, contrary to the expressed opinion of this high authority before mentioned, Dr. Taylor. During the last epidemic in this section of the State not more than fifty per cent. of the population over the age of three years escaped, and since then fifty per cent. of the remainder have been affected by this disease. Children are for some unexplainable reason not as liable to become infected as adults, and those under three years of age are largely exempt. No disease with which I have come in contact probably gives the patient the mental and bodily unrest that does influenza. Coming as it does generally with its first manifestation of a nasal catarrh or gastric irritation, followed within a few hours, or accompanied, and often really preceded by intense lumbar pains, aching with legs and head of soreness of whole body, chilliness, often amounting to a distinct chill; cough is almost always present in the respiratory variety, and often in the gastric form, sore throat and hoarseness in most of the severe cases. The mental faculties are acute, except as to the extreme nervousness and in the cases of active delirium. The systemic prostration is marked from the beginning in most cases—and the small irregular pulse is almost diagnostic. In fact, so far as the direct danger is concerned in an uncomplicated case, it is altogether to be expected from this condition of heart failure alone. This failure of the heart as before stated is accompanied by coexisting muscular and general bodily weakness. Profuse cold perspiration—danger signals warning us of the profound toxemia that is paralyzing all the forces of life. I have not found the sudamina so often stated as one of the constant symptoms, in a majority of my cases. But the tongue is always deeply coated, first generally white and deeply indented edges. Often dark brown offensive coating, especially on back part. In the gastric variety you more often find the red tongue than the white or brown. The pulse in my experience is high, generally from 110 to 130; and temperature from 100 to 104. In no disease is there so complete anorexia—all thought of food being nauseating, vomiting in many cases being a constant symptom. The bowels are not regular; sometimes being open, sometimes constipated. The urine is high colored and has the sweetish fever smell that is so constant in the continued fevers. From the history of the disease, as we see it, we might reasonably expect a very high death-rate, and yet such is not the case. Very few uncomplicated cases result fatally. However, in my opinion, there are few acute diseases that are to be so much dreaded as this. When you think for a moment of its complications—nearly all of which are immediate results of its prostrating effect upon the vital functions—among the most severe that we meet in the practice of medicine, first of the respiratory tract, pneumonia stands at the head of the list. So far as I have been able to find in post-mortem tables no death from lagrippe has been recorded that did not show either lobular or lobar consolidation of the lung. Laryngitis, bronchitis, congestion and œdæma of lungs, pleurisy, abscess and empyema, all being common complications. In the gastric varieties, the acute or subacute gastritis, the severe forms of diarrheas and dysentery. The bilious or "jaundiced" condition of the liver or severe hyperemia of that organ. All these of themselves go to make up a formidable list of human afflictions. And when we try to enumerate their sequelæ, we do not longer speculate at the gravity of this disease. When we have once realized the direct danger from this disease and its complications we are at least partly prepared to recognize its yet most dangerous features—the condition in which it leaves our patient—the things that so easily come after it—its sequelæ.

In no disease, that is of itself not dangerous, is the whole human organism left in a condition of such supreme state of exhaustion as in lagrippe, thus rendering the patient a most promising subject for the attack of any disease—the weakened nerve system during convalescence, carrying the patient into insanity, most often of the form of melancholia, though in my experience this has happened in only three cases. The condition of the eyes is such that in a large majority of cases it is weeks and even months before the patient can use them for reading any length of time—the functional disturbance often developing into an iritis, and even neuritis. Nasal catarrh, as might be expected, is a frequent sequel. A normal condition of the heart in severe cases is not regained for a period of less than three months, very often not until after six months or more—persistent vertigo attending your patient for months and months. Indigestion, gastric irritation and diarrhea often follow even mild attacks. Chronic nephritis is one of the most dangerous sequels to which the convalescent is specially liable. Otitis is perhaps third on the list of complications, and as a result many cases are left with impaired hearing. From the nature of the disease we would naturally expect a large percentage of cases to be rendered special subjects for after-attacks of pulmonary diseases. Hence we find many cases of consumption engrafted on these convalescents or following within a few months, weeks or years. In my own mind the great prevalence of tuberculosis in this country is directly traceable to the epidemic of influenza in 1891 and the cases we have had each year since. In my own section I have been able to satisfy myself beyond a doubt that this is the case. I have seen it suggested that the increase of appendicitis during the past fifteen years is more than likely one of the complications or sequels of this disease. When we come to the treatment of the disease there is much that might be said, but I shall confine myself to those things that I have personally used and in which I place my reliance. The diagnosis being comparatively easy, the character of the disease well known, the treatment should be a simple matter. In most adult cases a full dose of calomel (ten grains) should be given; five to ten grains of sulph. quinine with an equal amount of Dover's powder, accompanying this or immediately following a full dose of whisky or brandy. The quinine and Dover's to be repeated as needed, generally every three or four hours. The stimulant will be needed from four to six times a day. A hot lemonade is very grateful at any time. Antipyrin and phenacetine or acetanilid must be given if fever is very high.

One-quarter to half grain codea given for the aching and cough if other remedies do not at once relieve and repeated as needed. Strychnine I give in nearly all cases. Have used sparteine in only a few cases. The complications must be looked for and treated as they arise. All convalescents must be looked after carefully and tonic treatment given them. Hypophosphites with strychnine is safely given in nearly all cases. Iron, quinine and strychnine should be given in most cases. From the beginning of a case plenty of nourishment in the form of milk and eggs and animal broths should be given. In convalescence a generous diet should be insisted upon. For weeks and months good food, exercise in the open air, sports and pleasant recreations are indispensable. By thus carefully looking after our patients in convalescence we will save ourselves many disappointments and many valuable lives.

I make no excuse for bringing this subject to the attention of this society. I consider it one of very grave importance to every physician who is in the general practice of medicine. And while I may have offered nothing but what we all may know—if I shall cause this disease to be watched more closely and followed farther into its convalescence than is usually done with almost any other disease, I shall feel amply repaid for the time I have occupied.—*The Carolina Medical Journal, August.*

---

### THE PREVALENCE OF ABORTION.

The able and courageous exposure of the conditions existing in Paris in reference to criminal abortion and prevention of conception which Dr. Doléris publishes in this number of the *Annals,* shows that the evils which we have to deplore in this country, and perhaps especially in New England, are not limited to any one nation.

In Germany, also, abortion has lately become very prevalent, and seems to occupy the attention of the profession at present. In fact, there is not such professional unanimity there in condemning it as obtains in France and in this country. Under the name of "facultative sterilization" it is openly claimed that the proper limits of the right and duty of physician to interrupt or prevent pregnancy are not confined to conditions of health which would make the continuation of gestation dangerous to the life of the mother. Learned professors admit the necessity of inducing abortion when there is danger of establishment of a psychosis as as well as when one exists; when pregnancy plunges the woman into a condition of "inexpressible sadness," when pregnancy is repeated at very short intervals; one professor even goes so far as to advise the "sterilization" of the woman "each time that the advent of a new infant will have a disturbing influence on the life of the family."*

Of course abortion has been practised always and everywhere. The oath of Hippocrates contains a provision against it. Juvenal deplores how few children are born in gilded beds and berates the midwives of his time for their practice of inducing abortion.

The Esquimaux use a canula of leather with a pointed piece of whalebone inside it which can be thrust out by pulling on a strand of tendon, so as to puncture the membranes, and then retracted. In Turkish harems abortion is reduced to a fine art; the negro women use cotton root bark; the savage races trample on the pregnant abdomen.

The new phenomenon which Dr. Doléris emphasizes is the fact that what has always been condemned as a crime is now not only tolerated, but openly encouraged, taught, assisted.

It has been supposed that the inactivity of the legal authorities in this country and their failure to prosecute and punish abortionists was due to some inferiority of our police systems or our legal machinery and that "they do these things better in France."

It is well known that here there is no interference with abortionists as such, but that they are only prosecuted if a woman dies or if some of her next-of-kin call on the legal authorities in case her health has been ruined. In other words our police and courts only protect the woman although the law protects the fetus.

The old wording of the law only made it active for a woman "quick with child" and practically the people and the law-courts take the ground that "quick" means that the child is old enough for its motions to be felt, although an English judge has ruled that "quick with child is having conceived."

Now we find that in France and also in Germany the courts are unable or unwilling to root out criminal abortion. In Berlin

*Sarwey. Deutsche med Woch. 1905–N '8.

the papers are full of advertisements of the abortionists, and in spite of the police the hideous business goes on just as it does here.

What then are the causes of this sudden change in popular feeling and in legal prosecution?

It would seem that there were several all acting together.

1st. There is a general slackening of respect for religious authority which has condemned abortion as a sin and as murder of an unborn and unbaptized child.

The average man or woman will not recognize that an embryo of three to six weeks is a human being or has a soul or can claim any right at all. It requires a certain imagination to consider an ovum as a human being merely because it is fecundated, and this amount of imagination few people possess or cherish as against their own greater interests.

2d. There is a general feeling that the world is getting too full of people, that with the stamping out of epidemics, the infrequency of wars and improvements in hygiene the world is filling up so fast that there is no longer duty nor reason in being fruitful and multiplying in order to replenish the earth. The doctrines of Malthus are taking firm root, and if it were not for prevention of conception and abortion the overpopulation would long ago have become a pressing question.

With the diffusion of comfort and the rise in standard of living, people are forced, as by the iron hand of fate, to limit their families or go under in the struggle for survival.

3d. There is in truth a grève du ventre, a "strike" of the womb. It is a part of the emancipation of woman, and like divorce it has come to stay.

It is no use to say to woman that God says, "in sorrow shalt thou bring forth children." She answers that it is rubbish, that God never said it and that she *won't*, there now.

The woman's point of view seems to be that she has been enslaved long enough. That now she is free, and that her body belongs to herself and no one else. That no woman ought to endure sexual intercourse unless she is disposed thereto, that if she does not love a man she ought not to live with him and is justified in getting a divorce, that if "by mistake" she gets pregnant, her physician ought to immediately "help her" out of her "difficulty." That there ought to be no children born who are not wanted in the family; that it is vulgar and indecent to have more children than can be properly brought up and educated or more than three at any rate; that the function and duty of a husband is to pay bills and make money and have an automobile and take his wife about and dress her well and not bother her with "nasty" allusions to his sexual desires, on pain of her divine displeasure.

Verily times are changed and what the poor old world is going to do about it passes our comprehension.

We would humbly suggest to young doctors contemplating matrimony, however, that in a forgotten part of a neglected book they will find a description of the kind of woman they would do well to find, even the virtuous woman of the book of Proverbs, for although she is not like the "new" woman, there are still such women to be found in the world, and now as in old times their price is far above rubies.—*Editorial Annals of Gynecology and Pediatry, August, 1905.*

---

### THE PROPHYLAXIS AND TREATMENT OF YELLOW FEVER.*

BY F. FORCHHEIMER, M.D., CINCINNATI, OHIO.

Of the ultimate *causa causans* of this disease we are as yet in ignorance. Of its mode of transmission we have acquired at least some very definite knowledge. To D. Carlos Finlay, of Havana, must be credited the first suggestion that the disease is conveyed by means of the mosquito, *Stegomyia fasciata.* A board appointed for the investigation of this disease in Cuba, and composed of Dr. Walter Reed, Dr. James Carroll, Dr. A. Agramonte, and Dr. Jesse W. Lazier, conclusively proved the truth of Finlay's suggestion. How Lazear gave up his life as a result of mosquito inoculation "in the cause of science and humanity" is a story now too well known, even among the general public, to need recapitulation here.

*An article forming, in substance, the chapter on Yellow Fever from the author's forthcoming work on The Practice of Medicine.—Ed. St. L. M. R.

That mosquito inoculation is the only way in which fever can be transmitted must be considered open to question. This can be decided only after the specific germ has been found and its whole natural history has been laid bare. But the practical results of the acceptance of the mosquito theory have been so far-reaching in Cuba, where the disease was formerly endemic, and which country was in a perpetual state of being quarantined against by the United States, that yellow fever has been practically exterminated there, and we have even lived to see Cuba quarantining against Florida, and at the present moment against New Orleans, where an epidemic of serious proportions, even though not comparable as yet in the extent and rapidity of its progress to those of past times, is in progress. Much of this result is due to the indefatigable work of the board before mentioned, and especially to its president, the late Dr. Reed; but great credit is also due to the intelligent co-operation of the surgeon-general of the army at that time, Dr. George M. Sternberg, whose research work in regard to this disease is well known. The present condition of Cuba may be looked upon as an achievement in scientific prophylaxis the like of which has never been accomplished for any other disease. According to this view, the prophylaxis of yellow fever hinges on the prevention of mosquito bites, and it has proved eminently satisfactory for the extermination of the endemic form of the disease.

It is splitting hairs to discuss whether yellow fever is contagious, as it is immaterial, for practical purposes, whether fomites themselves, or the mosquitoes contained within them, propagate the disease, and, under all circumstances, a yellow fever patient is necessary for the production of the disease in others. That contagion by direct contact alone with patients is rarely, if ever, followed by the disease has been repeatedly shown. In the epidemic of 1878, when over thirty cases were imported from Memphis to Cincinnati, eight of which I saw, not a single case of infection occurred. On the other hand, Gallipolis, Ohio, which had previously had local epidemics, again suffered because of three refugess, but the acceptance of the mosquito theory easily explains this curious fact.

*Prophylaxis.*—The general acceptance of the principle that yellow fever is non-contagious, would effect much good in the presence of an epidemic, because it would strengthen individual resistance by allaying fear, would do away with needless barbarity, such as shotgun quarantine, and would benefit both patient and physician, by removing the senseless dread of them that has hitherto prevailed. It is a great pity that in the present epidemic this stage of general enlightenment is far from having been reached; indeed, the state of panic seems to be as far in advance of that in former epidemics as the fulminant character of the epidemic is, notwithstanding its undoubtedly severe type, behind it.

*General Prophylaxis.*—The general principles of sanitation go without saying. The important special measures consist in the destruction of mosquitoes, and particularly, of course, the *Stegomyia fasciata*, and the preservation of patients and well people against their bites.

The destruction of the adult females may be effected to some extent individually in closed spaces, but they are more readily destroyed by fumigation, especially with sulphur. The destruction of the larvæ depends principally upon the detection and abolition of their breeding places. These are always collections of water, large or small. Several methods have been used, the best being the establishment of good drainage. In the case of malaria, also a mosquito-transmitted disease, this of itself has been able to overcome the prevalence of the disease. Another method of destroying the larvæ is by suffocation. In order to get air they must come to the surface of the water; this has led to the sprinkling on the surface of the water of various oils, but principally petroleum; this should be used not only on fresh water collections, but also where diluted salt water collects. This method has been largely used in this country for the purpose of exterminating all kinds of mosquitoes; so far, however, the reports of its efficacy are contradictory.

Measures must also be taken to prevent the biting of yellow fever patients by the stegomyia. The victims of the disease should be strictly isolated, and measures should be taken, by the use of wire netting, to prevent access of mosquitoes to them, for until the stegomyia has sucked the blood of a yellow fever patient, it is powerless to convey the disease. When large hospitals can be erected, special wards should be used; if this is not possible, the bed containing the patient should be surrounded by wire gauze, of which the meshes should be 2.5 mm. wide. Mosquito netting has been shown to be comparatively inefficient and has been largely discarded. All doors and windows should be screened, and the latter furnished with strong springs. The room, also, should be darkened, for, like most insects, the mosquito follows rays of light.

Mosquitoes may be carried by fruits, hay, or fomites, and as is well known they have been shipped from Italy to London for experimental purposes, and malaria has then been produced by them in the human body. What is possible with the anopheles is probably equally so with the stegomyia.

From these considerations it follows that persons coming from an infected region should be kept under observation until five days have elapsed after the possibility of the infection, and their bedding, clothing, and baggage should be disinfected. It was the elation following the far-reaching discoveries that prompted the statement in Cir. No. 5 from the Headquarters department of Cuba (1901) that "they [the fomites] need not be subject to any special disinfection," qualified somewhat afterwards by the recommendation that they should not be removed from infected rooms until formaldehyde had been used, in order to destroy the infected mosquitoes. We are not, as yet, entitled to such perfect confidence, and for prophylactic purposes no chances of any sort should be taken. Long experience has shown that in this disease fumigation with sulphurous acid is followed by the best results. Infected ships, dwelling-houses, rooms, railway cars, in fact all places where individuals have congregated, should thus be disinfected. Formaldehyde seems to have been very valuable in Cuba.

*Individual Prophylaxis.*—While the only definite thing we have is the mosquito theory, the results of centuries of experience should not be neglected. The patient should be dealt with as in malarial disease. Every effort should be made to increase the resistance of the body. As in all infectious diseases, hygienic measures are of enormous value; good normal food, pure drinking-water, and healthy surroundings; overexertion of all sorts, physical and psychical, should be avoided; in the tropics all work should be done in the coolest parts of the day; exposure to the sun should under all circumstances be avoided. Alcohol does harm, and in the tropics should not be used at all.

Sanarelli's serum, bactericidal, has been used for prophylactic puposes; it seems to be followed by better results in prophylaxis than in therapeusis, in so far as any beneficial results at all are to be looked for. Rego Cesar has recommended the use of small doses of arsenous acid, gramme 0.0005 to 0.0004 (grains 1-30 to 1-15) four times daily, continued for months.

*Treatment.*—As there is no specific treatment for yellow fever, we can only follow the directions laid down by those who have had most experience in this disease, especially as the results obtained by applying the ordinary rules for the treatment of such fevers as occur in northern climates, are anything but good. The first thing for successful treatment is good nursing, the next is a physician who will not attempt too much. Every patient, if only a suspect, should immediately receive an efficient cathartic of castor oil or calomel, the action of which is to be hastened by injections. For intestinal antisepsis benzonapthol or corrosive sublimate has been recommended, to follow the purgation. The patient is to be kept in bed, warmly covered, and no food of any sort should be given for the first four or five days. For the thirst, a one-half per cent. solution of sodium bicarbonate in Vichy water may be given; it can also be relieved by rectal injections of sterilized water, given every six to eight hours. This is the routine treatment that seems to give the best results, as testified to by all those American physicians who saw a great number of cases in Cuba.

*Symptomatic Treatment.*—Symptomatically, the following have been found useful: For the fever, sponging with aromatic vinegar or baths when necessary. No antipyretics; quinine should never be used unless there is a malarial complication. For anuria, poultices to the lumbar region, the caffeine preparations, enteroclysis. For sleeplessness, chloral hydrate by rectal injection, icebag to the head for headache or delirium. For hemorrhage, ergotin or local measures. For collapse, alcohol or other stimulants.

In the severest cases, urema and cholemia, due to organic changes in the kidneys and the liver, resist all treatment; it is asserted that the routine method recommended prevents the development of these very severe forms.

*Convalescence.*—The patient should not be allowed to get up too early; never until a week has elapsed from the termination of the secondary fever. When albuminuria is present the patient must also be kept in bed, as in nephritis. The diet should be carefully controlled; the opinion is common that, as in typhoid, relapses as produced by carelessness, overfeeding and the introduction of indigestible food should be avoided. If the patient is to be sent away from home, a sufficient time must elapse for all evidences of the disease to disappear; his strength and resistance should be nearly normal, otherwise relapse or secondary infection may occur.

---

### PAIN AND VOMITING IN DISEASES OF THE STOMACH.*

BY H. M. READ, M.D.,

SEATTLE, WASH.

From the standpoint of the physician the stomach should receive more consideration and, now that so many are operating, or attempting to operate, upon this organ for troubles more or less plain or obscure, we should redouble our efforts to diagnose the existing disease before subjecting the patient to a severe surgical operation the final result of which, under the most favorable conditions and regardless of the disease, must for a long time remain in doubt.

The general practitioner or surgeon, as a rule, does not make a sufficiently careful investigation of stomach diseases before applying treatment. Any fair-minded physician or surgeon will admit the truth of this statement, and, while now and then, it will require an exploratory operation to determine the disease, most of them could be cleared up if we could give more time to the study of the case, and in so doing apply the various laboratory tests and study the clinical symptoms, not separately, but together with the clinical history. I have in the past, and so have you, told many patients they had "stomach trouble," or "dyspepsia," or "catarrh

*Read before the King County Medical Society, Seattle, Wash.

of the stomach," and told them to be "careful of their diet," and "take this," and now and then they got better and sometimes they came back for some more of the same brand of foolishness, and more often came no more.

Those happy days of complacency are rapidly fading and to solve the problems of internal medicine, and successfully diagnose medical and surgical cases we must apply the master word used in the wonderful address delivered by Dr. Osler.

First, pain is the almost ever-present symptom in many diseases ot the stomach and is expressed in many ways. Alone it is not of much value, but when linked with vomiting and certain physical conditions, with the findings in the analysis of test meals, etc., as a rule, it has a special significance for each separate disease.

Vomiting is not so reliable a symptom, for it is often absent in serious disease, but it is present often enough so as to be a valuable aid when taken in connection with pain and certain clinical conditions.

I will pass the age of infancy and early childhood, when organic stomach trouble is comparatively rare, to early youth and thence to old age during which time it is common.

A very interesting group of cases are those of young females and males at the chlorotic age of from twelve to twenty years, and almost entirely confined to females; Bettman says 90 per cent. In this group pain is a very common symptom of organic stomach disease. However, now and then a case will occur in which the vomiting of blood or actual perforation of the stomach may be the first symptom of disease. This group of cases is admirably described by Bettman, in his recent article, as acute primary ulcer, to mark them from chronic or secondary types. The pain is severe and comes after taking any kind of solid or semi-solid food. It persists for some time, unless relieved by vomiting, and is associated with certain pain points or tender spots, which nearly all recent authorities agree are generally present in ulcers of the stomach, either acute or chronic, the first place a small area in the epigastric region just to the left of the medium line, the second on the left of the spine from the tenth to the twelfth dorsal vertebra, or both. These spots are usually very tender, the one in front being more sensitive to a very light than a deep pressure. Their presence so far can only be explained as due to some reflex irritation to the roots of the spinal nerves. In the above group of cases, occurring in young people, the anemic condition with pain on eating and relief by vomiting, or a pain that is relieved by rest in bed, with a milk diet, in the presence of the pain points described, is undoubtedly a case of gastric ulcer; for, while many chlorotic young women and girls vomit and have stomach pain, this is usually cured by a course of iron and nourishing food, and rest in bed with a milk diet does not influence that condition, and then again, in cases of ulcer in these individuals they are made worse by full diet and the administration of iron, if these are resorted to before the ulcers are cured. Vomiting of blood will occur in the majority of all cases of ulcer of the stomach some time during the course of the disease.

Recently orthoform is coming into use as a valuable aid in the diagnosis of, as well as in the treatment of gastric ulcer, especially in acute or recent cases; in ulceration it nearly always stops the pain very promptly. In anemic young individuals who have stomach pain and vomiting, which are not due to ulceration, the pain points will be absent and the pain will not be relieved by orthoform and milk diet. (The dose of orthoform is from 8 to 15 grs.) The examination of the stomach contents in the group of foregoing cases will not be of much value, for an excess of hydrochloroic acid is not a symptom of acute ulcer.

When a person vomits blood we should be sure that it was vomited, for severe or prolonged vomiting may produce a capillary hemorrhage which usually amounts to only streaks of blood; then, again, the blood might come from other sources as the throat, lungs, gums, or nose, and in young women we should at least think of vicarious menstruation from this portion of the body when the normal function has been suppressed.

The above group of cases seems to me a very important one, for they are often treated as cases of simple chlorosis. In connection with ulcer cases in general, pain is an important and almost constant factor, coming on anywhere from a few minutes to an hour or two after taking food, is very severe, is usually relieved by vomiting which will occur involuntarily in one-half of the cases, and a majority of these cases will vomit blood sometime during the course of the disease. There will usually be the pain points. The pain is usually aggravated by taking solid food and the patient will soon lose weight. A strict milk diet, with rest in bed and the administration of orthoform, will soon relieve the pain in any case of ulcer, and an analysis will usually show excess of hydrochloric acid, unless it is an old case associated with stenosis and dilatation or is a very recent acute case, and we should always rely very much upon the effect of absolute rest in bed, freedom from all excitement, and strictly milk diet, with the internal administration of orthoform to aid in making our diagnosis more secure. The pain produced by simple hyperchlorhydria is very similar to that of ulcer, but is not associated with vomiting, is usually relieved by taking solid food and is always very promptly relieved by large doses of sodium bicarbonate, and the pain points are absent.

Gastric erosion is described by Einhorn, as one of the most painful afflictions of the stomach, is always, or nearly always, associated with some vomiting, is made worse by any kind of food, liquid or solid, and in the vomitus will be found pieces of the gastric mucous membrane which have been exfoliated, and these cases are promptly relieved or cured by milk diet and nitrate of silver lavage.

Cases of supposed hyperchlorhydria which do not get well under a diet of milk and eggs, with large doses of sodium bicarbonate after meals, are undoubtedly something more than simple hyperchlorhydria, for it must be remembered that sodium carbonate will even relieve for a time the pain of ulcer, when the same is associated with excess of hydrochloric acid and when given in conjunction with a milk diet. This relief, however, is temporary and other conditions will help to clear the diagnosis and we should never forget that gastric ulcers are extremely chronic, and we should remember the regularity of the pain in one particular place after taking food, the presence of the pain points, and the vomiting.

Many neurotic conditions produce pain in the stomach, but in the neurasthenic, or the person with brain fag, or incipient brain, or spinal disease, the pain is different; for example, any kind of food may produce pain or vomiting in these cases at once, while in ulcer some time, even if very short, will elapse between the taking of food and the onset of the pain and vomiting. In nervous cases the temperature of the food makes no difference and vomiting does not relieve the pain, nor will milk and the administration of orthoform give relief; the pain points are not present, they do not usually lose flesh rapidly or much, nor do they generally vomit all the food taken, and an analysis will show normal hydrochloric acid.

We must always remember that angina from arteriosclerosis may begin in the stomach instead of the chest and arms and radiate to the legs instead of the arms, or from there to the arms, and a pain of this character occurring in people past the middle of life with a good history of alcoholism, syphilis, or hard work and exposure, alone or combined, is suggestive of angina, where there is arteriosclerosis. This character of pain should call to our minds the above suggested conditions, even if the pain is associated with or followed by nausea, as it not infrequently is, for attacks of angina pectoris or angina abdomina not infrequently are precipitated by a full meal. I have personally observed this condition a number of times. Syphilis alone will not infrequently produce pain, more or less constant and severe, beginning in the region of the stomach and radiating to the back and shoulders. This does not refer to the initial symptoms of tabes dorsalis, but comes in acute cases during the febrile stage. I have now one case under treatment who suffered very much as above described, and the pain was very promptly relieved as soon as the case was brought under the influence of mercury. We should always bear in mind that tabes dorsalis very frequently begins with a severe pain in the stomach, even with vomiting, but these cases can be easily differentiated by attention to the clinical signs and symptoms, including, of course, the chemical work, it being only necessary to remember that in only very rare cases we may have syphilitic ulceration and inflammation of the stomach and the history of the case should at once remind us of the condition. In neurotic women with pain and vomiting we must look for perverted habits, especially those of swallowing clay, chalk, hair, etc.

Gallstone colic, especially when associated with organic stomach disease, is entirely different from the pain of stomach disease alone. It is right-sided, is not relieved but usually made worse by vomiting, and often continues for several hours after there is absolutely nothing in the stomach; the pain radiates to the back and shoulders and umbilicus, and an analysis between the attacks will show a normal condition of the stomach secretions.

Acute pancreatitis might possibly be confounded with acute ulceration or acute gastritis, but here the pain is usually to the left, the onset is sudden, temperature high, vomiting does not relieve the pain, but rather makes it more severe, the shock is much more profound, and the general clinical picture far more serious than that of stomach disease, especially perforation of stomach or duodenal ulcer or an advanced or early malignancy. Primary cancer of the pancreas, besides being very rare, would be easily differentiated by the absence of many of the clinical signs of stomach disease.

Vomiting of undigested food, without pain or much pain, several hours after meals, with a diminished or absent hydrochloric acid, or loss of flesh, usually indicates a dilated stomach with pyloric obstruction, with or without ulceration.

It is difficult to diagnose duodenal ulcers, the attacks of pain being right-sided, radiating to the stomach and back, and often so closely simulates gallstone colic that it has been operated upon for such by some no less skillful than Mayo. The vomiting in ulcer of the duodenum, when it does occur, apparently bears not much relation to the time of eating, and vomiting does not stop or relieve it, and if there is tenderness it is to the right instead of the left of the medium line and the gastric ulcer pain points are wanting.

The pain of acute or chronic gastritis is diffuse, of an aching character, and in chronic cases the stomach bloats, the tongue is covered with a foul coating, (in gastric ulcer the stomach is red) and an analysis shows a large amount of mucus, diminished hydrochloric acid, and a large amount of lactic and other acids, which result from fermentation of food; that is, the total acidity will be high with a marked diminution of free and hydrochloric acid.

The most confusion arises in the diagnosis of ulcer from cancer, and of course we all know the frequency with which carcinoma begins in an ulcer and, in some cases just on the border line, the symptoms and clinical signs may be identical. However, we do not often see these cases, for when they come to us the malignancy has so far advanced that we need only to remember a few important points, and where a diagnosis in any case of stomach trouble can not be made with reasonable certainty, an exploratory laparotomy should be made.

One point in connection with carcinoma should be this, I believe, that in 90 per cent. of all cases of cancer, and in more than that later on, the free hydrochloric acid is absent from the stomach contents. I know this percentage is disputed by authority that is excellent, still I believe it to be not too high. In the majority of cases, as soon as malignancy becomes pronounced, the breath becomes foul and the tongue covered with a more or less foul coating. In ulcer hydrochloric acid is increased in about 90 per cent. of all cases and those cases of malignancy reported in which the acid is normal or increased, when other signs of ulcer are found, are undoubtedly cases of early malignancy engrafted upon an ulcer.

It should be remembered that the pain of cancer does not bear much relation to the taking of food, although the vomiting may, and the dietary treatment heretofore mentioned, as applied to ulceration, will have very little, if any, permanent influence upon the pain. The pain of cancer is not relieved by vomiting. While the presence of a palpable tumor at the cancer age, in the presence of pain and a marked anemia with progressive loss of weight, makes the diagnosis pretty sure, we should not forget that an old perforating ulcer at the pylorus and the adhesions may cause a tumor and obstruction, simulating cancer very closely.

In a case of cancer near the pylorus vomiting is always present, emaciation rapid and continuous and a marked grade of anemia will be present. The amount of blood vomited is not so great as in ulcer, and is usually mixed with food, mucus and ferment acids, forming the well-known coffee-ground vomit.

In any suspected disease of the stomach wherein the symptom of vomiting is the most prominent, we should always think of the various reflex cases, and I would especially mention chronic appendicitis.

It would be impossible to cover so large a field as is contained within the boundaries of this important and much neglected subject by writing one small paper. I have not mentioned the more recent clinical methods employed in the diagnosis of stomach disease, including the occult blood test as applied to the stools in suspected ulcerative or malignant disease, but have tried to confine myself to the two most important symptoms of stomach disease.

The surgical department of our profession is doing a good deal to stir the medical men to study their cases more carefully, and the "up-to-date" medicine man can render much of the operating unnecessary by treating properly diagnosed cases more intelligently.

---

### EXTRAGENITAL CHANCRES.

BY ABRAHAM L. WOLBARST, M.D., NEW YORK.

Extragenital chancres are by no means uncommon, and but little can be added to the literature of the subject. The following cases, however, present such unusual features that their recording may not be without justification.

CASE 1.—M. W., bartender, aged 22 years, native American, of good education, single, general health good, presented himself in August, 1901, with what appeared to be a mild form of phlegmon of the left index finger. The nail and its margins were not involved. There was a moderate amount of pain, slight fluctuation, and a slight rise of temperature. Though the clinical picture of phlegmon was not complete, local treatment having been without avail, an incision was made under local anesthesia (ethyl chloride spray). The incision at once revealed the suspicious character of the infection. Instead of pus, we drew about half a drachm of colorless serum. The walls of the wound were of a greenish gray color, granular in appearance, and seemingly dry and hard. The curette was applied rather superficially, and the wound loosely packed with wet bichloride gauze. Within two weeks after the incision all doubts of the character of the wound were set aside when a typical roseola appeared all over the body. The patient was put under appropriate antisyphilitic treatment, and continued his treatment fully three years. He has since married and at this writing his wife is pregnant.

How the infection took place is easily explained. Bartenders in removing glasses from the bar, usually pick up three or four glasses at one time, taking hold of them by the tips of the fingers inserted inside the glasses. Presumably the glass picked up by the patient with his left index finger had been used by a syphilitic with mouth or tongue ulcerations, and he was thereby infected through an abrasion in the skin.

The fact that a positive diagnosis was not made until the appearance of the secondary rash, is another point in favor of regarding every finger wound that is suspicious in the least manner as a possible chancre.

CASE 2.—H. K., merchant, 30 years old, unmarried, general good health, presented himself in October, 1900, with a diagnosis of hemorrhoids, made by a physician whom he had visited. He had been under the latter's care about ten days without relief. In fact, the condition was growing worse all the time. Subjectively the symptoms were those of external hemorrhoids, of a mild type.

Examination revealed a superficial ulceration at the junction of the anal mucous membrane with the skin, about half an inch in diameter, the surface of which was smooth and shining. It was quite soft and slightly tender to the touch. Its margins were fairly regular, but not sharply outlined against the adjacent skin. Chancre was suspected, and a placebo administered pending the appearance of corroborative symptoms.

Within two weeks the ulceration assumed a typical appearance. It was about an inch in diameter, margins distinctly defined and infiltrated, and presented an unmistakably specific character. The diagnosis of chancre was confirmed when the classic eruption appeared twenty days later. The man was given antisyphilitic treatment, but discontinued his treatment when the external symptoms disappeared. He has not been heard from since.

Careful questioning revealed a most shocking state of affairs,

This man, like many others of his set, was in the habit of indulging in unnatural practices. Given a syphilitic tongue and an anal fissure ever so slight, and the source of infection is revealed.

CASE 3.—J. B., 3 years old, son of Italian immigrants, but a few months in this country. Had never been ill. General appearance healthy. Mother brought him because of a marked phimosis, which she discovered the day previously. The child also seemed to have pain on micturition for several days.

Examination revealed a profuse discharge from the urethra, containing many gonococci. The urine contained pus and was quite bloody and was passed only after much straining and pain. The prostate was enlarged and tender, and the left epididymis was somewhat thickened and exquisitely tender to the touch. In addition to this severe posterior urethritis, the child presented a well-defined specific lesion, situated about one inch below the umbilicus, in the median line. It had every characteristic of the classic chancre, even to the violaceous color and the infiltration. There was a general glandular enlargement throughout, and the inguinal glands were tender, presumably because of the gonorrhea. There were no secondary manifestations.

On inquiry, the mother said that she had noticed the sore about one week previously, but the child not complaining of pain or discomfort, she paid no attention to it. A placebo was administered and three weeks later the general roseola appeared.

A most searching examination of the parents, brothers, and sisters of the child revealed not the least trace of the source of infection, and we can but surmise on the manner in which this child was infected with syphilis and gonorrhea simultaneously. There can be no doubt that the infection was the result of the vicious attacks of degenerates upon children, which of late have appeared in large numbers in many quarters.

CASE 4.—L. M., salesman, aged 26 years, married six months, health good. Wife two months pregnant. For two weeks previous to his first visit in December, 1902, he was troubled with a peculiar sensation on the posterior surface of his left thigh, at the junction of its upper and middle thirds. He felt no pain, but described the sensation as one of distinct numbness of the skin at that point. He also observed that the skin was somewhat thickened and harder than the surrounding tissues. Had applied ointments locally without relief.

On examination a characteristic and typical hard chancre was noticed at the spot indicated, which was followed some four weeks later by the classic roseola. Under appropriate treatment he made an uneventful recovery. It may be noted, in passing, that his wife gave birth to a healthy child at full term seven months later.

As to the source of infection, it is most probable that an infected water-closet seat may have been responsible. No other source of infection could be suggested after the most careful inquiry.

Case 5.—B. H., salesman, aged 30 years, American, unmarried, and in previous good health. Presented himself in January, 1903, with a malculo-papular eruption all over the body, general glandular enlargement, and a small erosion on the buccal mucous membrane. He had noticed the eruption while taking a bath that morning.

Thorough search of the genital organs revealed no trace of the initial lesion, and the patient could not recall having had any lesion anywhere on the body that might have served as the point of entry of the disease. Recalling the history of Case 2, I directed the inquiry along the lines indicated by that experience, and found, at the junction of the anal mucous membrane with the skin, the remains of a typical hard chancre. Investigation revealed a history of infection similar to that of Case 2, with all its revolting features.

Case 6.—B. A., mechanic, aged 22 years, health sound, presented himself in March, 1904, with a bluish purple, localized area, about an inch in diameter, and irregular outline, situated on the inner aspect of the right thigh, over the center of Scarpa's triangle. A diagnosis of possible chancre was made, and was later confirmed when secondary symptoms appeared. In this case the mode of infection could not be determined or even surmised.

